# Effect of stride length on the running biomechanics of healthy women of different statures

**DOI:** 10.1186/s12891-023-06733-y

**Published:** 2023-07-24

**Authors:** Aravind Sundaramurthy, Junfei Tong, Adhitya V. Subramani, Vivek Kote, Michael Baggaley, W. Brent Edwards, Jaques Reifman

**Affiliations:** 1 Department of Defense Biotechnology High Performance Computing Software Applications Institute, Telemedicine and Advanced Technology Research Center, United States Army Medical Research and Development Command, FCMR-TT, 504 Scott Street, Fort Detrick, MD 21702-5012 USA; 2 The Henry M. Jackson Foundation for the Advancement of Military Medicine, Inc, Bethesda, MD 20817 USA; 3 Human Performance Laboratory, Faculty of Kinesiology, University of Calgary, Calgary, AB T2N 1N4 Canada; 4 The McCaig Institute for Bone and Joint Health, Cumming School of Medicine, University of Calgary, Calgary, AB T2N 1N4 Canada

**Keywords:** Individualized models, Musculoskeletal injury, Stature, Stride length, Tibial stress fracture

## Abstract

**Background:**

Tibial stress fracture is a debilitating musculoskeletal injury that diminishes the physical performance of individuals who engage in high-volume running, including Service members during basic combat training (BCT) and recreational athletes. While several studies have shown that reducing stride length decreases musculoskeletal loads and the potential risk of tibial injury, we do not know whether stride-length reduction affects individuals of varying stature differently.

**Methods:**

We investigated the effects of reducing the running stride length on the biomechanics of the lower extremity of young, healthy women of different statures. Using individualized musculoskeletal and finite-element models of women of short (N = 6), medium (N = 7), and tall (N = 7) statures, we computed the joint kinematics and kinetics at the lower extremity and tibial strain for each participant as they ran on a treadmill at 3.0 m/s with their preferred stride length and with a stride length reduced by 10%. Using a probabilistic model, we estimated the stress-fracture risk for running regimens representative of U.S. Army Soldiers during BCT and recreational athletes training for a marathon.

**Results:**

When study participants reduced their stride length by 10%, the joint kinetics, kinematics, tibial strain, and stress-fracture risk were not significantly different among the three stature groups. Compared to the preferred stride length, a 10% reduction in stride length significantly decreased peak hip (*p* = 0.002) and knee (*p* < 0.001) flexion angles during the stance phase. In addition, it significantly decreased the peak hip adduction (*p* = 0.013), hip internal rotation (*p* = 0.004), knee extension (*p* = 0.012), and ankle plantar flexion (*p* = 0.026) moments, as well as the hip, knee, and ankle joint reaction forces (*p* < 0.001) and tibial strain (*p* < 0.001). Finally, for the simulated regimens, reducing the stride length decreased the relative risk of stress fracture by as much as 96%.

**Conclusions:**

Our results show that reducing stride length by 10% decreases musculoskeletal loads, tibial strain, and stress-fracture risk, regardless of stature. We also observed large between-subject variability, which supports the development of individualized training strategies to decrease the incidence of stress fracture.

## Background

Musculoskeletal injuries, such as stress fracture, pose a recurrent health threat to military personnel and civilian athletes. For example, stress fracture accounts for approximately 1.6 million injuries per year in the U.S. military [1] and is the leading cause of Soldier lost duty days during Army basic combat training (BCT) [2]. In fact, the incidence of stress fractures in BCT recruits is 18 times higher than that of experienced military personnel [3]. Among civilian athletes, the occurrence of stress fracture varies between sports, with track and long-distance runners experiencing the highest incidence [4]. Interestingly, it has been consistently reported that, compared to men, women are more susceptible to stress fracture during both BCT (9% women vs. 3% men) and in professional and collegiate training (10% women vs. 7% men) [5].

Risk factors for stress fracture can be categorized as non-modifiable (e.g., race, stature, and sex) and modifiable (e.g., stride length, training volume, and load carriage) [6]. Several studies have examined the impact of both types of risk factors [7–11]. For example, Bulathsinhala et al. explored the influence of race and ethnic origin on injury risk among active-duty women in the U.S. Army. Their findings indicated that, compared to Black women, White women exhibited the highest incidence of stress fractures, followed by American Indian/Native Alaskan, Hispanic, and Asian women [7]. Similarly, an individual’s stature is also suspected to affect the incidence of stress fracture, although its effect is not conclusive [12, 13]. For instance, while examining the risk factors that influence orthopedic injuries in young female recruits who underwent basic military training, Moran et al. observed a higher incidence of stress fractures in taller as compared to shorter women [12]. Wentz et al. suggested that this difference could be attributed to the long bone structure of taller individuals, which may experience a higher degree of bending and an increase in bone strain [5]. However, in our previous study involving young, healthy women running at a constant speed of 3.0 m/s, we found that individuals with a large stature experienced higher joint forces and moments, but not higher tibial strain or stress-fracture risk, than individuals with a short stature [14]. In contrast, Jones et al. noted that shorter women are more susceptible to pelvic stress fracture because, during group-marching and running activities, shorter women are typically placed in the rear of the formation and, to keep pace with their taller counterparts, they overstride, which increases the mechanical load placed by the adductor and hamstring muscles on the pubic rami and the likelihood of pelvic stress fracture [13].

Modifiable factors, such as training volume, load carriage, and stride length during running, provide an opportunity to intervene and lower injury risk. For example, in our previous work, we investigated the stress-fracture risk for women of different statures while running with no load or a 22.7-kg load at a constant speed of 3.0 m/s, and found that, when compared to the no-load condition, the 22.7-kg load increased the tibial strain and stress-fracture risk among women of short and medium stature but not in tall women [14]. Several studies have shown that reducing stride length while maintaining a constant running speed induces changes to the joint kinematics [15, 16] and decreases peak values of the joint kinetics [15, 17, 18], ground reaction force (GRF) [15], tibial strain, and tibial stress-fracture risk [19]. For instance, Heiderscheit et al. analyzed motion-capture data from healthy adult volunteers (men and women) who ran on a treadmill at their preferred speed for five different step rates (preferred, ± 5%, and ± 10%) and showed that a 10% increase above the preferred step rate at a constant speed decreases the energy absorbed at the knee and hip joints [15]. In addition, they showed that increasing the step rate decreases the peak GRF as well as the hip adduction and hip and knee flexion angles during the stance phase [15]. A reduction in the hip adduction angle may decrease the risk of iliotibial band syndrome [20], and a reduction in the stance-phase knee flexion angle is associated with a decrease in the peak patellofemoral force [21]. Hafer et al. examined the running biomechanics of men and women who ran over ground on a 30-m runway at their self-selected constant speed with a preferred stride rate or a 10% higher stride rate, and found that the higher stride rate resulted in a decrease in knee extension moment, which is known to decrease the force transmitted across the knee [17]. Furthermore, Edwards et al. predicted that reducing the stride length by 10% for men running over ground at a self-selected constant speed decreases the risk of stress fracture by 31% [19].

The objective of this study is to assess the effect of reducing the stride length on the running biomechanics of young, healthy women of different statures. To this end, we developed *individualized* musculoskeletal and finite-element (FE) models using experimental data collected from 21 women of three stature groups (short, medium, and tall). Using the individualized computational models [14] and a previously developed probabilistic stress-fracture risk-prediction model [22], we calculated the joint kinematics and kinetics at the lower extremity and the tibial strain and stress-fracture risk for each participant when running at their preferred stride length and when they reduced their stride length by 10%. We hypothesized that stride length changes the running biomechanics of young, healthy women and that such changes depend on stature. Specifically, we hypothesized that reducing the stride length would decrease joint forces and moments, tibial strain, and tibial stress-fracture risk for women of all stature groups. Additionally, we hypothesized that the magnitude of these reductions would differ depending on the stature of the participant.

## Methods

### Study data

To develop our computational models, we leveraged experimental data of 21 young, healthy women (18–21 years old) in three stature groups (N = 7 for each of short, medium, and tall) from a previous study, in which we investigated the effects of stature and load carriage on the biomechanical responses at the tibia [14]. As in the previous study, here we set the stature (i.e., height) criteria as less than the 35th percentile (short; 1.48–1.60 m), between the 35th and 70th percentile (medium; 1.60–1.66 m), and greater than the 70th percentile (tall; 1.66–1.78 m) of the U.S. female Soldier population [23]. We purposely recruited participants in three different groups to approximately represent each tertile of Army personnel. All participants were self-reported experienced treadmill runners and had not experienced any injuries that would limit their physical activity 3 months before enrollment in the study. For each participant, we collected their age, mass, height, body mass index, foot length, and body fat percentage (Table 1). Furthermore, we collected computed tomography (CT) images of each participant’s left tibia, at a resolution of 0.49 × 0.49 mm^2^ and with a slice thickness of 0.63 mm, using a General Electric Discovery Scanner (General Electric Medical System, Milwaukee, WI). During scanning, we included a calibration phantom (QRM, Moehrendorf, Germany) with known hydroxyapatite concentrations in the field of view.

**Table 1:** Anthropometric characteristics of 20 young, healthy women

Stature	Age (years)	Mass (kg)	Height (m)	Foot length (m)	Body fat (%)	BMI (kg/m^2^)
Short (N = 6)^*^	19.7 (1.0)	54.1 (5.4)	1.55 (0.03)	0.23 (0.01)	16.4 (2.4)	22.5 (2.4)
Medium (N = 7)	19.3 (0.7)	60.5 (3.9)	1.63 (0.02)	0.24 (0.01)	18.5 (4.0)	22.7 (1.9)
Tall (N = 7)	20.0 (1.1)	65.2 (4.7)	1.74 (0.02)	0.25 (0.01)	19.6 (1.5)	21.6 (1.7)
***p*** **value**	0.153	**0.002**	**< 0.001**	**0.002**	0.147	0.540

Data are presented as means (one standard deviation). A bold *p* value indicates the parameter is significantly different at the 0.05 level among the three stature groups, based on an analysis of variance test. BMI: body mass index. *One participant was excluded from the computational analysis due to loose marker placement during the study, which affected motion tracking

Each participant completed two running trials in a randomized order at a constant speed of 3.0 m/s on an instrumented treadmill (Bertec Corporation, Columbus, OH), with one trial at their preferred stride length and the second trial at a stride length of approximately 10% less than their preferred stride length. We chose a 10% reduction based on literature review, which indicates that runners are able to successfully incorporate a stride-length reduction of up to 10% into their running routines through gait retraining [17]. To achieve a 10% reduction in stride length while maintaining a constant running speed of 3.0 m/s, we asked participants to increase their step frequency by 10% using the beats of a metronome as a reference. As previously described [14], we collected motion-capture data at 200 Hz using an eight-camera motion-analysis system (Vicon Nexus, Centennial, CO), where we tracked 42 retroreflective markers bilaterally on each participant’s arms, trunk, pelvis, thighs, shanks, and feet, and synchronously collected force-platform data at 1,000 Hz. For each trial, we collected 20 s of data after the participant reached a steady-state stride at 3.0 m/s, thus providing a sufficient number of strides (> 20) to obtain consistent stance and swing durations for both preferred and − 10% conditions from which to select a representative stride.

The study protocol was approved by the University of Calgary Conjoint Health Research Ethics Board and by the Office of Human Research Oversight at the U.S. Army Medical Research and Development Command, Fort Detrick, MD. We obtained written informed consent from each participant before enrollment in the study.

### Selection of a representative stride

After acquiring the motion-capture data, we analyzed the 20-second recordings from each participant’s running trial and chose one representative stride because we cannot aggregate the recordings and use their mean or median values for a computational model. To select a representative stride for the preferred condition, we followed the method of Sangeux and Polak [24]. First, we identified the start and end points of each stride in the 20-second recordings by setting a threshold of 25 N for the vertical GRF. Next, we resampled the vertical GRF time history of each stride so that strides of different durations were represented by 100 vertical GRF data points, computed the median vertical GRF time history, and ranked each vertical GRF time history based on how close it was to the median time history. Finally, we chose the stride that was closest to the median as our representative stride.

To select a representative stride for the − 10% condition, we followed the first three steps described above, where we identified the start and end points of each stride by setting a vertical GRF threshold of 25 N, resampled each vertical GRF time history so that each stride was represented by 100 data points, and computed the median stride from the resampled vertical GRF time histories. However, in the final step, we calculated the stride length of each vertical GRF time history by multiplying the stride duration and the running speed. Then, instead of choosing the stride closest to the median, we chose the stride with a stride-length reduction closest to 10% of the representative stride chosen for the preferred condition. If multiple strides had the same stride-length reduction, then we chose the one that was closest to the median.

### Individualized musculoskeletal and finite-element models

We provided a detailed description of the individualized musculoskeletal and FE models in our previous work [14]. Briefly, for the individualized musculoskeletal model, we extracted the subject-specific tibial geometry from the CT scans using 3-Matic (Materialise, Leuven, Belgium) and morphed the tibial geometry into a generic female musculoskeletal model available in the AnyBody system (AnyBody Technology, Aalborg, Denmark). Then, we scaled the other body segments of the generic model based on the anthropometric measurements of each participant, such as foot length, mass, height, and body fat percentage. Finally, we employed an optimization scheme that minimized the errors between the markers defined in the model and those tracked in the experiment. Once optimized, using the marker-trajectory data for the representative stride, we computed the body motion (i.e., the joint angular changes throughout the entire body), including the kinematics of the hip, knee, and ankle joints. Then, we estimated the kinetics of the hip, knee, and ankle joints by performing an inverse dynamics analysis and normalizing the GRF and the joint reaction forces by body weight, and the joint moments by body mass, for each participant running with their preferred stride length and a 10% reduction in stride length.

For the individualized FE model, first, we acquired the subject-specific tibial geometries from the CT scans. Next, using Hypermesh software (Altair Engineering, Inc., Troy, MI), we discretized the tibial geometry using 10-noded quadratic tetrahedral elements, with an average element size of 3.0–3.5 mm. Then, we assigned a linear elastic and isotropic Young’s modulus (E) for each element, which we computed using the Hounsfield units of the CT scan. Based on Young’s modulus, we categorized the elements as intramedullary tissue (E < 6 MPa), trabecular bone (6 MPa ≤ E < 8 GPa), or cortical bone (8 GPa ≤ E). As the bone and tissue components have different Poisson’s ratios, we assigned a ratio of 0.325 to trabecular and cortical bone elements and 0.167 to intramedullary tissue elements [25]. Finally, we applied the muscle forces, joint forces, and joint moments computed using the musculoskeletal model for the representative stride as the input loading condition for the FE model. Specifically, we coupled the muscle and ligament insertion points from the individualized musculoskeletal model with the outer surface of the tibial FE mesh. We created 171 couplings for each individualized FE model and performed FE analysis using Abaqus 2019 (Dassault Systèmes, Vélizy-Villacoublay, France). We calculated the von Mises strain for each cortical bone element and derived the 90th percentile von Mises strain of the tibial cortical bone due to the loading from the representative stride, for each of the two conditions.

### Probabilistic stress-fracture risk-prediction model

We predicted the risk of tibial stress fracture using a probabilistic model that accounted for bone fatigue damage, adaptation, and repair [26]. A detailed description of the model is provided in our previous work [22]. Briefly, the risk-prediction model used the tibial strain estimated by the individualized FE model as input to determine the bone’s fatigue life based on a S-N curve obtained from a beam-bending experiment on the human tibial bone [27]. To incorporate bone adaptation, we adjusted the tibial strain for each day by multiplying it by a strain adaptation ratio using beam-theory equations, assuming a bone deposition of 4 μm/day [28]. Given the number of loading cycles per day, adjusted with a bone repair rate of 26 days, the model then predicted the tibial stress-fracture risk as a function of the number of exercise days [26].

We evaluated the risk of stress fracture for two training regimens relevant to military recruits and recreational marathon runners. In the first regimen, we evaluated a 10-week BCT regimen [29], wherein we converted marching cycles during each day of BCT into equivalent running cycles and added the daily running cycles to obtain the total running loading cycles per day. Then, we defined a representative week by averaging and prorating the running cycles into five training days (1.7 km/day) and two rest days (no running). We repeated the representative week 10 times to simulate a 10-week BCT regimen. In addition, we also investigated the effects of running with the preferred and − 10% stride-length conditions when we doubled the running distance from 1.7 km/day to 3.4 km/day, five days a week for each of the 10 weeks of BCT, and estimated the stress-fracture risk. In the second training regimen, we evaluated a 20-week recreational training (RT) regimen for marathon runners [30] by implementing a weekly running program consisting of five training days (9.6 km/day) and two rest days (no running). We repeated this weekly schedule 20 times to simulate a 20-week RT regimen. For all regimens, to calculate the daily loading cycles for each participant, we divided the running distance per day by their individual stride length. We then utilized the number of daily loading cycles and the corresponding tibial strain to estimate the individualized risk of tibial stress fracture at the end of the training regimen for each participant.

### Statistical analysis

Prior to participant recruitment, we performed a power analysis and determined that seven subjects per group were sufficient to observe group-based differences with a statistical power of 75% and a significance level of 10%. We computed the sample size with group means and standard deviations of the leg stiffness and peak vertical GRF from a previous study where participants ran on an instrumented treadmill at an average speed of 3.3 m/s while carrying a load [31]. For the anthropometric characteristics, we performed an analysis of variance to determine statistically significant differences among the short, medium, and tall stature groups. To determine the impact of stride length (preferred and − 10%) and stature (short, medium, and tall) on running biomechanics, we developed linear mixed-effects models for various dependent variables (e.g., joint moments, joint kinematics, and tibial stress-fracture risk). Specifically, the full model included three fixed categorical effects (i.e., stride length, stature, and stride length-stature interaction) and a random intercept that accounted for within-subject dependence. For each dependent variable, we calculated the significance of the interaction term using the Wald *F*-test, with degrees of freedom adjusted using the Kenward-Roger method [32]. If the interaction term was statistically significant, we performed a *post hoc* Tukey’s pairwise comparison between the preferred and − 10% groups [33] for each of the three stature groups separately, based on the estimated marginal means, with a Holm-Bonferroni adjustment for *p* values. If the interaction term was not statistically significant, we removed it from the model and re-evaluated the main effects (i.e., stride and stature) using the Wald *F*-test. If we found that the stride was statistically significant, we performed a *post hoc* Tukey’s pairwise comparison on the two stride lengths [33] for each of the three stature groups separately. Because we only had two stride conditions, when stride alone was significant, we did not perform a *post hoc* comparison. For the tibial stress-fracture risk, we applied a log transformation to the raw data prior to analysis because the data were not normally distributed. We presented all data as means (one standard deviation) unless otherwise noted. We performed statistical analyses with an alpha level of 0.05 with the RStudio v1.4 statistical software using *lme4*, *lmerTest*, and *emmeans* functions.

## Results

During our analysis, we excluded one woman from the short-stature group due to loose marker placement during motion tracking. In total, our study included six women of short stature and seven women each in the medium- and tall-stature groups. We performed an analysis of variance on the anthropometric measurements and found no significant differences in age, body fat percentage, or body mass index (Table 1). In contrast, among the three stature groups, we found significant differences in mass (*p* = 0.002), height (*p* < 0.001), and foot length (*p* < 0.002). Table 2 shows the average stride length achieved by each stature group for the preferred and − 10% conditions.

**Table 2:** Stride lengths estimated from the representative stride for the preferred and −10% conditions

Stature	Stride length (m)	
	Preferred	-10%
Short (N = 6)	2.06 (0.15)	1.85 (0.14)
Medium (N = 7)	2.06 (0.12)	1.86 (0.11)
Tall (N = 7)	2.12 (0.09)	1.90 (0.08)

Stride lengths were calculated from the representative stride for short, medium, and tall women while running with their preferred stride length (Preferred) and a 10% shorter stride length (-10%) relative to the preferred stride length. Data are presented as averages over each stature group (one standard deviation)

### Joint kinematics

**Fig. 1:**
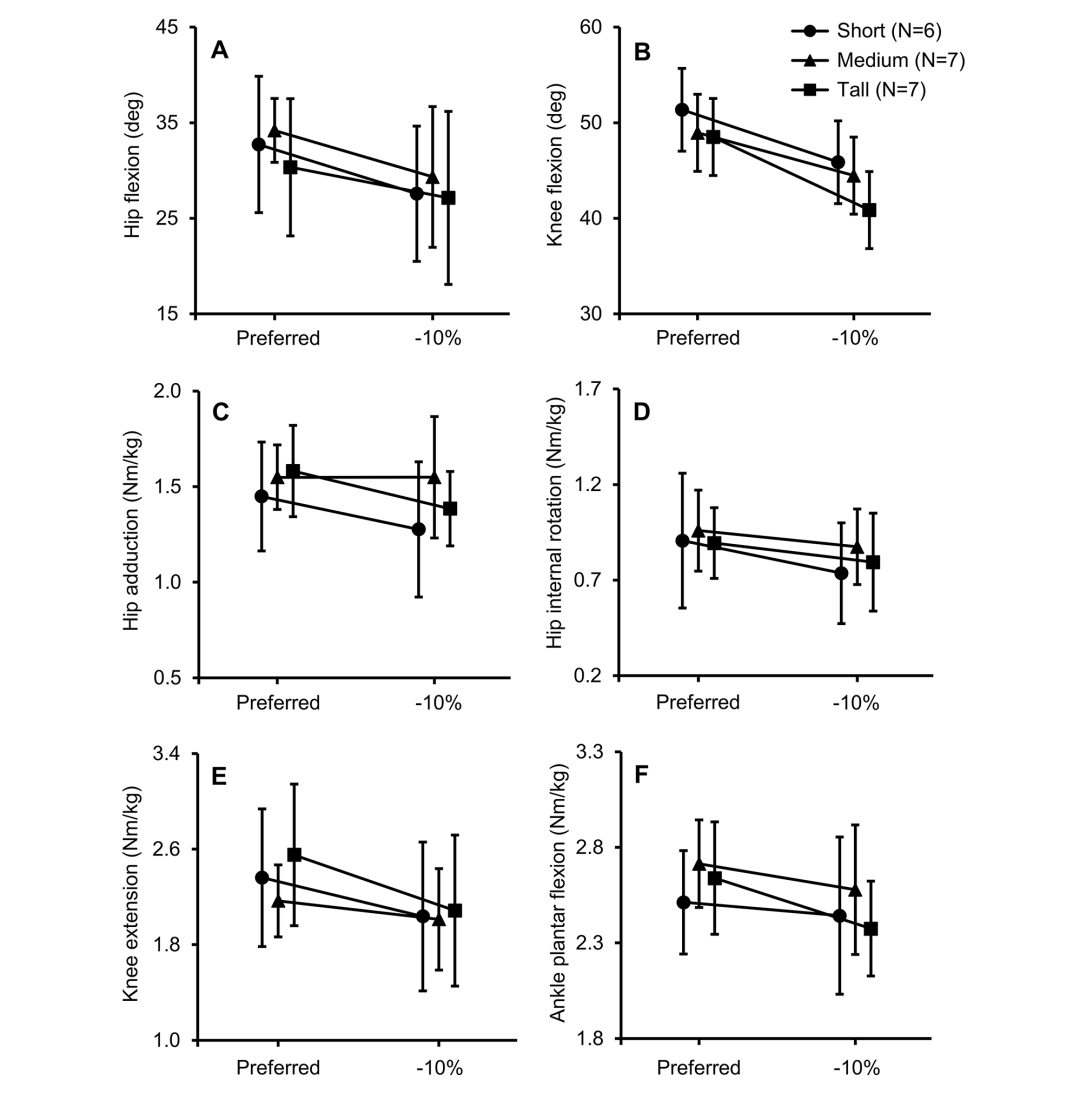
(**A**) peak hip flexion angle during stance, (**B**) peak knee flexion angle during stance, (**C**) peak hip adduction moment, (**D**) peak hip internal rotation moment, (**E**) peak knee extension moment, and (**F**) peak ankle plantar flexion moment for short (circle), medium (triangle), and tall (square) women while running with their preferred stride length (Preferred) and a 10% shorter stride length (-10%) relative to their preferred stride length. Error bar: 95% confidence interval. A linear mixed-effects model using the Wald *F*-test indicated significant differences between stride lengths (*p* < 0.05) for all parameters

Table 3 shows the mean (one standard deviation) of the joint kinematics computed using the individualized musculoskeletal model, for each of the three stature groups. Reducing the stride length had a statistically significant effect on the peak hip (*p*_*stride*_ = 0.002) and knee (*p*_*stride*_ < 0.001) flexion angles during the stance phase (Table 3; Fig. 1). However, stride length did not affect the peak hip adduction angle (*p*_*stride*_ = 0.362) or the ankle dorsiflexion angle during initial contact (*p*_*stride*_ = 0.410). In contrast to stride length, our statistical analysis showed that neither stature nor the interaction between stature and stride length influenced the hip, knee, and ankle kinematic variables (Table 3). Hence, we combined the stature groups and reported the results for the two variables that showed a statistically significant difference between preferred and − 10% conditions in Table 4. Specifically, a 10% reduction in stride length decreased peak hip flexion angle by 14% and peak knee flexion angle by 12%.

**Table 3:** Joint kinematics and peak joint moments for the preferred and −10% conditions

	Short		Medium		Tall		*p* value		
	Preferred	-10%	Preferred	-10%	Preferred	-10%	Stride	Stature	Stride-stature
**Joint kinematics (degrees)**									
Peak hip adduction	9.4 (5.9)	7.5 (4.0)	7.1 (2.4)	6.2 (3.1)	4.7 (3.4)	5.5 (3.7)	0.362	0.261	-
Peak hip flexion^S^	32.7 (6.8)	27.6 (6.7)	34.2 (3.3)	29.3 (7.4)	30.3 (7.1)	27.1 (9.1)	**0.002**	0.683	-
Peak knee flexion^S^	51.3 (7.0)	45.9 (8.5)	48.9 (4.2)	44.5 (3.5)	48.5 (4.2)	40.9 (4.6)	**<0.001**	0.375	-
Ankle dorsiflexion^IC^	14.3 (7.8)	12.4 (11.0)	15.8 (5.1)	17.0 (4.3)	16.9 (4.8)	13.6 (4.7)	0.410	0.608	-
**Peak joint moment (Nm/kg)**									
Hip adduction	1.4 (0.3)	1.3 (0.3)	1.5 (0.2)	1.5 (0.3)	1.6 (0.2)	1.4 (0.2)	**0.013**	0.398	-
Hip internal rotation	0.9 (0.3)	0.7 (0.2)	1.0 (0.2)	0.9 (0.2)	0.9 (0.2)	0.8 (0.2)	**0.004**	0.730	-
Knee extension	2.4 (0.5)	2.0 (0.6)	2.2 (0.3)	2.0 (0.4)	2.5 (0.6)	2.1 (0.6)	**0.012**	0.656	-
Ankle plantar flexion	2.5 (0.2)	2.4 (0.4)	2.7 (0.2)	2.6 (0.3)	2.6 (0.3)	2.4 (0.2)	**0.026**	0.448	-

Estimated values of joint kinematics and peak joint moments for short, medium, and tall women while running with their preferred stride length (Preferred) and a 10% shorter stride length (-10%) relative to the preferred stride length. Data are presented as averages over each stature group (one standard deviation). To determine the impact of stride length and stature on the running biomechanics, we used a statistical model that had stride, stature and its interaction as main effects. Because the interaction term was not significant for any of the variables, we did not report *p* values for the interaction term in the table. The reported *p* values are from a model with only stride and stature as its main effects. A bold *p* value indicates a statistically significant main effect. S: stance phase; IC: initial contact

**Table 4:** Variables that showed a statistically significant effect on stride length

	Stride length	
	Preferred	-10%
**Peak joint kinematics (degrees)**		
Hip flexion^S^	32.7 (5.9)	28.0 (7.5)
Knee flexion^S^	49.5 (5.1)	43.6 (5.8)
**Peak joint moment (Nm/kg)**		
Hip adduction	1.5 (0.2)	1.4 (0.3)
Hip internal rotation	0.9 (0.2)	0.8 (0.2)
Knee extension	2.3 (0.5)	2.0 (0.5)
Ankle plantar flexion	2.6 (0.3)	2.5 (0.3)
**Peak joint reaction force (BW)**		
Hip	9.4 (1.5)	8.1 (1.3)
Knee	12.6 (1.2)	11.1 (1.1)
Ankle	12.6 (0.9)	11.6 (1.5)
**Tibial strain (με)**		
	5,007 (867)	4,421 (922)
**Tibial stress-fracture risk (%)** ^*^		
BCT	2.3 (1.3–4.1)	0.9 (0.5–1.8)
RT	12.9 (7.8–21.2)	5.5 (3.0–10.2)

With the exception of the tibial stress-fracture risk, data are presented as means (one standard deviation) averaged for all 20 women. *We transformed the tibial stress-fracture risk to its original scale using the antilog function and presented it as the average value (95% confidence interval). S: stance phase, BCT: basic combat training, RT: recreational training

### Joint moments

Table 3 also shows the mean (one standard deviation) of the peak joint moments based on inverse dynamics computations of the individualized musculoskeletal models. Again, we found that the joint moments did not depend on the stature of the participants or the interaction between stride length and stature, whereas a 10% reduction in stride length resulted in a significant reduction in the peak values of hip adduction (*p*_*stride*_ = 0.013), internal rotation (*p*_*stride*_ = 0.004), knee extension (*p*_*stride*_ = 0.012), and ankle plantar flexion (*p*_*stride*_ = 0.026) moments (Table 3; Fig. 1). When we combined the stature groups, we observed reductions in the peak hip adduction moment (7%), hip internal rotation moment (11%), knee extension moment (13%), and ankle plantar flexion moment (4%), as illustrated in Table 4.

### Joint reaction forces

**Fig. 2:**
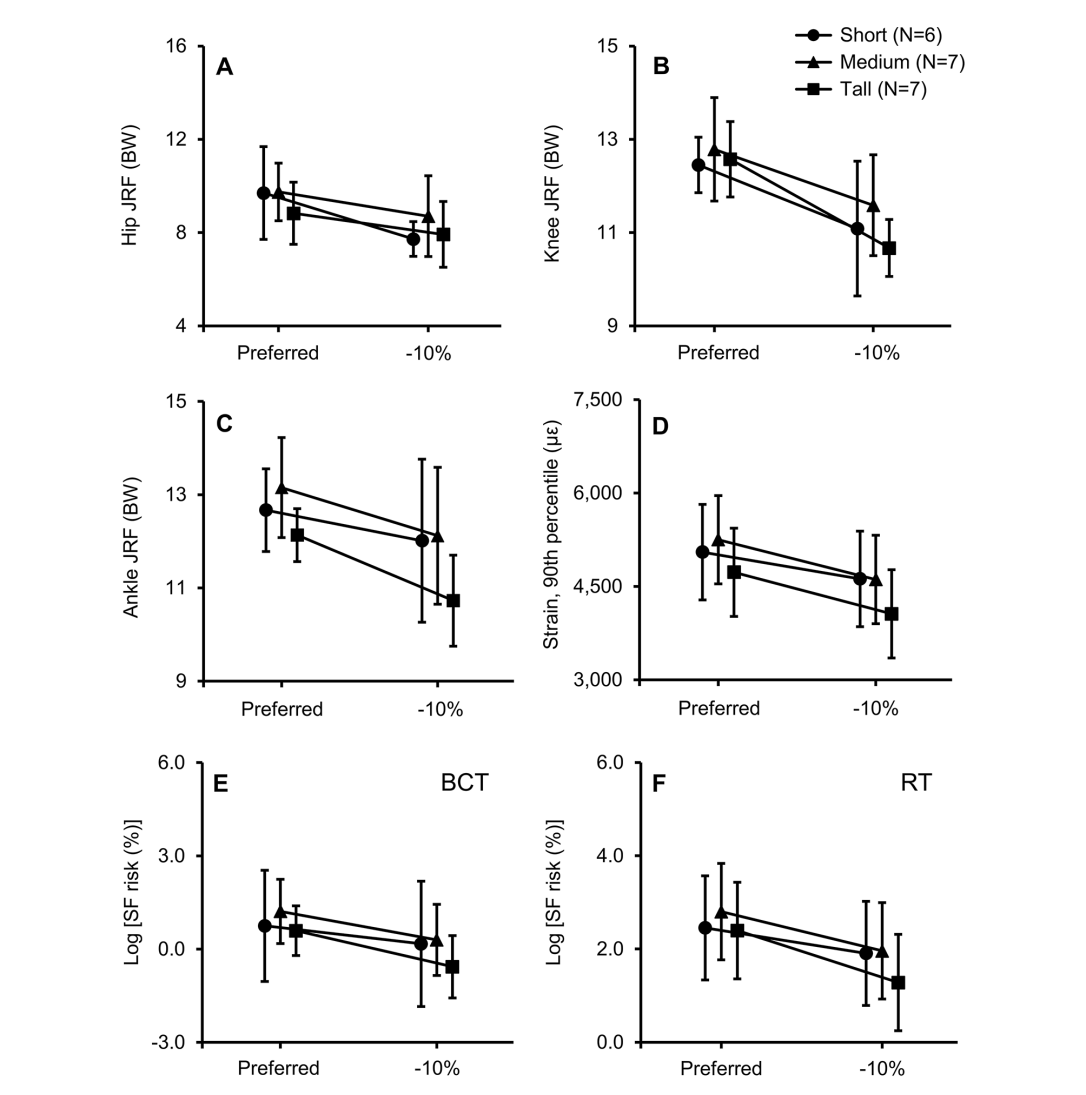
(**A**) peak hip joint reaction force (JRF), (**B**) peak knee JRF, (**C**) peak ankle JRF, (**D**) 90th percentile strain, (**E**) log-transformed stress-fracture (SF) risk for a 10-week basic combat training (BCT) regimen, and (**F**) log-transformed SF risk for a 20-week recreational training (RT) regimen for short (circle), medium (triangle), and tall (square) women while running with their preferred stride length (Preferred) and a 10% shorter stride length (-10%) relative to their preferred stride length. Error bar: 95% confidence interval. A linear mixed-effects model using the Wald *F*-test indicated significant differences between stride lengths (*p* < 0.05) for all parameters

Table 5 shows the mean (one standard deviation) values of the peak joint reaction force and the peak vertical GRF based on inverse dynamics computations of the individualized musculoskeletal models. Similar to the joint kinematics, neither the stature nor the interaction between stature and stride length influenced the joint reaction forces. Conversely, reducing the stride length by 10% significantly decreased the joint reaction forces in the hip (*p*_*stride*_ < 0.001), knee (*p*_*stride*_ < 0.001), and ankle (*p*_*stride*_ < 0.001) (Table 5; Fig. 2). Specifically, we observed decreases in the hip (14%), knee (12%), and ankle (8%) joint reaction forces (Table 4). Although neither stature nor stride length had a significant influence on the vertical GRF (Table 5), we observed a consistent decrease when participants reduced their stride length by 10%.

**Table 5:** Peak JRF, peak vertical GRF, strain, and SF risk for the preferred and −10% conditions

	Short		Medium		Tall		*p* value		
	Preferred	-10%	Preferred	-10%	Preferred	-10%	Stride	Stature	Stride-stature
**Peak joint reaction force (BW)**									
Hip	9.7 (1.9)	7.7 (0.7)	9.7 (1.2)	8.7 (1.7)	8.8 (1.3)	7.9 (1.4)	**< 0.001**	0.501	-
Knee	12.4 (0.6)	11.1 (1.4)	12.8 (1.1)	11.6 (1.1)	12.6 (0.8)	10.7 (0.6)	**< 0.001**	0.459	-
Ankle	12.7 (0.8)	12.1 (1.1)	13.1 (1.1)	12.1 (1.5)	12.1 (0.6)	10.7 (1.0)	**< 0.001**	0.102	-
**Peak vertical ground reaction force (BW)**									
	2.5 (0.2)	2.4 (0.3)	2.4 (0.1)	2.3 (0.2)	2.4 (0.1)	2.3 (0.1)	0.074	0.550	-
**Tibial strain (με)**									
	5,050 (1,247)	4,621 (1,293)	5,250 (795)	4,611 (824)	4,727 (539)	4,059 (619)	**< 0.001**	0.498	-
**Tibial stress-fracture risk (%)** ^*^									
BCT	2.1 (0.3–12.6)	1.2 (0.2–8.9)	3.4 (1.2–9.4)	1.3 (0.4–4.2)	1.8 (0.8–4.0)	0.6 (0.2–1.5)	**< 0.001**	0.562	-
RT	11.6 (2.4–56.4)	6.7 (1.1–41.1)	16.4 (6.7–40.6)	7.1 (2.3–21.9)	11.0 (5.1–23.5)	3.6 (1.3–9.6)	**< 0.001**	0.566	-

Estimated peak joint reaction force (JRF), peak vertical ground reaction force (GRF), tibial strain, and stress-fracture (SF) risk for a simulated basic combat training (BCT) regimen and a recreational training (RT) regimen for short, medium, and tall women, while running with their preferred stride length (Preferred) and a 10% shorter stride length (-10%) relative to their preferred stride length. With the exception of the tibial SF risk, data are presented as averages over each stature group (one standard deviation). To determine the impact of stride length and stature on running biomechanics, we used a statistical model that had stride, stature and its interaction as main effects. Because the interaction term was not significant for any of the variables, we did not report *p* values for the interaction term in the table. The reported *p* values are from a model with only stride and stature as its main effects. A bold *p* value indicates a statistically significant main effect. *We transformed the tibial SF risk to its original scale using the antilog function and presented it as the average value (95% confidence interval). BW: body weight

### Tibial strain and stress-fracture risk

**Fig. 3:**
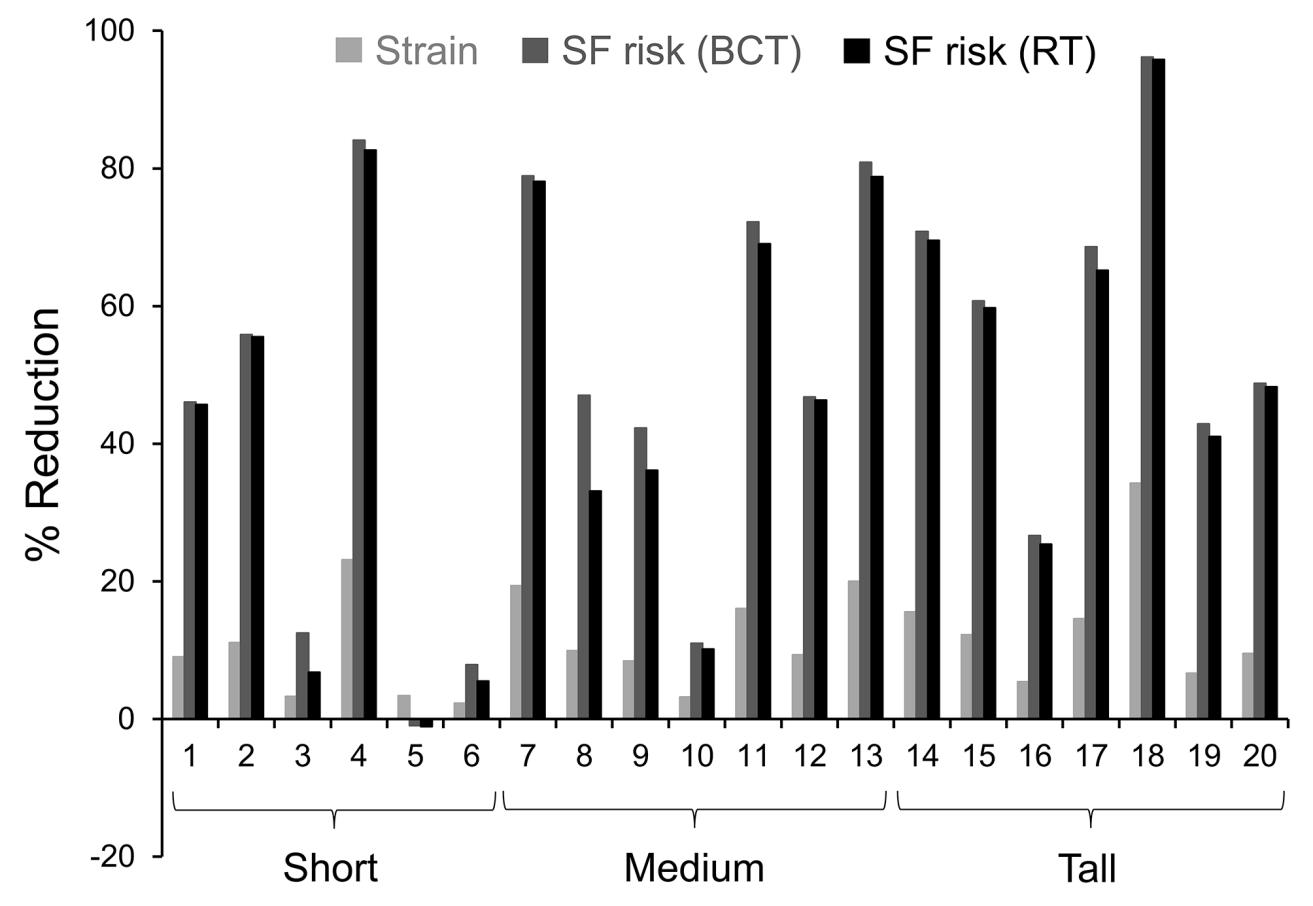
Subject-specific percentage reduction in tibial strain and stress-fracture risk for a 10-week basic combat training (BCT) regimen and a 20-week recreational training (RT) regimen due to a 10% reduction in stride length as compared to a preferred stride length. The strain reductions reflect the output of the individualized finite-element model, while the risk reductions reflect the output of the probabilistic model, for each running regimen

Table 5 also shows the tibial strain and stress-fracture risk computed based on the individualized FE models and the probabilistic stress-fracture risk-prediction model, respectively. As in the analysis discussed above, only a reduction in stride length caused significant decreases in the tibial strain (*p*_*stride*_ < 0.001) and stress-fracture risk for both the BCT (*p*_*stride*_ < 0.001) and RT (*p*_*stride*_ < 0.001) regimens (Table 5; Fig. 2). Specifically, when participants reduced their stride length by 10%, the tibial strain decreased by 12%. In addition, on average, the stress-fracture risk decreased by 61% for the BCT regimen and 57% for the RT regimen (Table 4). Figure 3 shows the percentage reduction in tibial strain between the two stride-length conditions computed by the FE model for each participant. The figure also shows the reduction in stress-fracture risk for each participant at the end of the simulated 10-week BCT and 20-week RT regimens. While the reduction varied from subject to subject, it was consistent across participants (except for participant 5) and independent of stature. Among the 20 participants, we observed strain reductions ranging from 2 to 34%, and risk reductions ranging from negligible to 96%.

## Discussion

The goal of our study was to quantify the impact of stride length on the running biomechanics of young, healthy women of different statures. To this end, we obtained the anthropometric measurements and tibial scans for women of three different statures, i.e., short (N = 6), medium (N = 7), and tall (N = 7), and collected motion-capture data while they ran at their preferred stride length and with their stride length reduced by 10%. Then, for each participant, we developed individualized musculoskeletal and FE models and quantified the risk of tibial stress fracture for running regimens representative of a 10-week U.S. Army BCT and a 20-week RT for marathon runners. In partial agreement with our hypothesis, we found that reducing the stride length decreased the joint moments, joint reaction forces, tibial strain, and tibial stress-fracture risk. However, in contrast to our hypothesis, these changes did not depend on the stature of the participants.

Both peak hip and knee flexion during the stance phase decreased significantly (14% and 12%, respectively) when the participants reduced their stride length by 10% (Tables 3 and 4; Fig. 1). Heiderscheit et al. [15] and Wang et al. [16] observed a similar decrease in the peak value of knee flexion during the stance phase, but not hip flexion, when participants increased their step rate by at least 5%. Heiderscheit et al. only observed a decrease in the peak hip flexion when the step rate increased by 10% [15], implying that knee flexion is more sensitive to an increase in the step rate (or a decrease in the step length) than hip flexion during the stance phase. A reduction in the peak knee flexion may decrease the patellofemoral joint reaction force in addition to reducing the external joint force and the demands on the musculature [21].

Joint moments decreased by 4 to 13% with a reduction in stride length (Tables 3 and 4; Fig. 1). Heiderscheit et al. observed similar decreases in the peak hip adduction, internal rotation, and knee extension moments when participants increased their step rate by 10% [15], and so did Hafer et al. for the peak hip adduction, knee extension, and ankle plantar flexion moments when subjects increased their stride rate by 10% [17]. We also observed decreases in hip (14%), knee (12%), and ankle (8%) joint reaction forces when participants reduced their stride length (Tables 4 and 5; Fig. 2). Thomas et al. observed a similar reduction in the hip, knee, and ankle joint reaction forces when the subjects decreased their stride length by 10% [18]. These decreases in the joint moments and reaction forces, especially in the knee and ankle, will reduce the loading on the tibia, resulting in a lower tibial strain and stress-fracture risk.

To assess the effect of stride-length reduction when running with and without load carriage, we compared our findings with studies involving changes in stride length with load carriage. For instance, Lobb et al. analyzed the lower-extremity biomechanics of men and women running over ground at a speed of 4.0 m/s based on their preferred stride length, as well as 15% longer and 15% shorter stride lengths, while carrying loads ranging from 20 to 35 kg. In agreement with our results, they found that the peak vertical GRFs are consistently lower when participants ran with a 15% shorter stride length, irrespective of load [34]. In contrast to our findings, when examining the running biomechanics of men and women for the same experimental conditions as Lobb et al., Brown et al. reported an increase in ankle flexion moment for all subjects running with a stride length that was 15% shorter or 15% longer than their preferred stride length, irrespective of load [35]. We attributed this discrepancy to body-movement adaptations required to maintain balance during running with a load, which suggests that the observed changes in lower-extremity biomechanics when running at a shorter stride length without a load may not necessarily translate to the condition in which participants run with load carriage.

We observed decreases in tibial strain and stress-fracture risk when the participants reduced their stride length (Table 5; Fig. 2). In particular, the 90th percentile of the von Mises strain in the tibia decreased by 12% (Table 4). Using this strain as an input to the stress-fracture risk-prediction model, we estimated the probability of tibial stress fracture for a 10-week U.S. Army BCT and a 20-week RT for marathon runners. After a 10-week BCT regimen, our predictions for the − 10% and preferred stride-length conditions indicated stress-fracture risks of 0.9% and 2.3%, respectively. These estimates are lower than the 8.0% risk reported by Knapik et al. in their study of women undergoing BCT [36]. We attributed this discrepancy to the fact that our study did not consider load carriage as well as other strenuous activities typically performed during BCT [29]. After a 20-week RT regimen, we estimated the stress-fracture risk to be 5.5% and 12.9% for the − 10% and preferred stride-length conditions, respectively. These findings align closely with the study by Kelsey et al. [37], who reported an incidence of tibial stress-fracture injury of 7.9% among 127 female cross-country runners with a weekly running distance of 55.5 km. On average, the 10% stride-length reduction decreased the risk of tibial stress-fracture injury by ~ 60% for both regimens (Table 4). Edwards et al. made a similar observation in a cohort of men, where the probability of stress-fracture risk decreased by 31% when participants reduced their stride length by 10% [19].

When comparing the estimated risks between BCT and RT, we observed a sixfold increase in the RT regimen, for both the preferred and − 10% stride-length conditions. We attributed this increase in risk to the increase in running distance between the two conditions (1.7 km/day for BCT vs. 9.6 km/day for RT). The longer distance results in a greater number of loading cycles, thereby increasing the risk of stress fracture. We also examined the impact of extending the BCT regimen by doubling the daily running distance to 3.4 km/day. This extension led to a substantial increase in risk for both the preferred and − 10% stride-length conditions, with a 115% increase compared to the original BCT regimen. These findings highlight the importance of considering running distance when assessing the risk of stress fracture, as longer distances over the same number of running days increase the risk.

For a fixed running distance, the number of loading cycles increases with a reduction in stride length. One could expect that such an increase in loading cycles would increase the metabolic cost of running and, in turn, cause muscle fatigue sooner than when running with a preferred stride length. Indeed, Heiderscheit et al. showed that increasing the step rate by 10% increases the rate of perceived exertion [15]. However, they conjectured that this increase in exertion could be due to an increase in cognitive focus required for adjusting the step rate rather than the metabolic cost of running. In support of this conjecture, Hamill et al. showed that increasing stride frequency by 10% does not significantly increase oxygen consumption or heart rate [38]. Therefore, as stated by Edwards et al. [19], decreasing stride length by 10% would not necessarily increase muscle fatigue.

Due to stride-to-stride variability, the differences we observed in the running biomechanics, as well as those in the calculated tibial strain and stress-fracture risk, between the preferred and − 10% conditions would be different for different selections of the representative stride. To minimize this variability, we used a systematic and reproducible procedure to select the representative stride for each condition [24]. While the percent reduction in tibial strain and stress-fracture risk varied from participant-to-participant, and these reductions would be different for different selections of the representative stride, a 10% shorter stride length resulted in a systematic reduction of strain and stress-fracture risk for all participants (Fig. 3). (Participant 5 was the only exception, where we observed a < 1% increase in the risk.) Interestingly, these reductions were independent of stature, with relative reductions in stress-fracture risk ranging from negligible to 84% for the short group, 10–81% for the medium group, and 25–96% for the tall group, for both the BCT and RT regimens.

Our study has limitations. First, we performed our running experiments at a constant running speed of 3.0 m/s on a level treadmill. Consequently, the conclusions may not apply to running at a different speed or on a graded treadmill. Second, we did not include complex three-dimensional motions at the knee and ankle joints, as we believe such inclusions would not change the conclusions regarding joint kinematics and kinetics. Third, similar to prior studies [14, 19], we assumed a uniform repair and bone adaptation process when predicting the tibial stress-fracture risk. Future studies may improve the risk prediction model by individualizing the bone adaptation and remodeling mechanisms and incorporating muscle fatigue. Finally, we conducted our experiments in a controlled laboratory environment using a treadmill and without load carriage. While this laboratory setup is not fully representative of military training conditions during BCT, we implemented it to minimize confounding factors and systematically delineate the effect of reducing stride length.

## Conclusion

We collected experimental data for young, healthy women of short, medium, and tall stature while they ran with their preferred stride length and with a 10% reduced stride length, and examined the impact of stride length and stature on their running biomechanics. We determined that reducing stride length by 10% reduced joint moments, joint reaction forces, and tibial strain. Moreover, for a simulated 10-week BCT regimen and a 20-week RT regimen, the stress-fracture risk decreased on average by 61% and 57%, respectively, ranging from negligible values up to 96% among 20 the subjects. However, these changes did not depend on the participant’s stature, suggesting that a 10% reduction in stride length led to similar beneficial changes in running biomechanics regardless of the participant’s height. We expect that our ability to examine running biomechanics at the individual level will help to quantify the risk of tibial stress fracture during military training and civilian recreational training, potentially guiding the development of individualized training programs to minimize injury risk [29].

## Data Availability

The data and the related analyses will be made available through a written request to the corresponding author, including a summary of the planned research.

## Abbreviations

BCT: Basic combat training
CT: Computed tomography
FE: Finite element
SF: Stress fracture
GRF: Ground reaction force
JRF: Joint reaction force
RT: Recreational training

## Declarations

### Disclaimer

The opinions and assertions contained herein are the private views of the authors and are not to be construed as official or as reflecting the views of the U.S. Army, the U.S. Department of Defense (DoD), or The Henry M. Jackson Foundation for the Advancement of Military Medicine, Inc. (HJF). Any citations of commercial organizations and trade names in this report do not constitute an official U.S. Army, DoD, or HJF endorsement of approval of the products or services of these organizations. This paper has been approved for public release with unlimited distribution.
